# Evaluating the performance of Bayesian and restricted maximum likelihood estimation for stepped wedge cluster randomized trials with a small number of clusters

**DOI:** 10.1186/s12874-022-01550-8

**Published:** 2022-04-13

**Authors:** Kelsey L. Grantham, Jessica Kasza, Stephane Heritier, John B. Carlin, Andrew B. Forbes

**Affiliations:** 1 School of Public Health and Preventive Medicine, Monash University, Melbourne, Australia; 2 Clinical Epidemiology and Biostatistics Unit, Murdoch Children’s Research Institute, Parkville, Australia; 3 Centre for Epidemiology and Biostatistics, Melbourne School of Population and Global Health, University of Melbourne, Carlton, Australia

**Keywords:** Bayesian inference, Cluster randomized trial, Intracluster correlation, Restricted maximum likelihood, Simulation study, Stepped wedge

## Abstract

**Background:**

Stepped wedge trials are an appealing and potentially powerful cluster randomized trial design. However, they are frequently implemented with a small number of clusters. Standard analysis methods for these trials such as a linear mixed model with estimation via maximum likelihood or restricted maximum likelihood (REML) rely on asymptotic properties and have been shown to yield inflated type I error when applied to studies with a small number of clusters. Small-sample methods such as the Kenward-Roger approximation in combination with REML can potentially improve estimation of the fixed effects such as the treatment effect. A Bayesian approach may also be promising for such multilevel models but has not yet seen much application in cluster randomized trials.

**Methods:**

We conducted a simulation study comparing the performance of REML with and without a Kenward-Roger approximation to a Bayesian approach using weakly informative prior distributions on the intracluster correlation parameters. We considered a continuous outcome and a range of stepped wedge trial configurations with between 4 and 40 clusters. To assess method performance we calculated bias and mean squared error for the treatment effect and correlation parameters and the coverage of 95% confidence/credible intervals and relative percent error in model-based standard error for the treatment effect.

**Results:**

Both REML with a Kenward-Roger standard error and degrees of freedom correction and the Bayesian method performed similarly well for the estimation of the treatment effect, while intracluster correlation parameter estimates obtained via the Bayesian method were less variable than REML estimates with different relative levels of bias.

**Conclusions:**

The use of REML with a Kenward-Roger approximation may be sufficient for the analysis of stepped wedge cluster randomized trials with a small number of clusters. However, a Bayesian approach with weakly informative prior distributions on the intracluster correlation parameters offers a viable alternative, particularly when there is interest in the probability-based inferences permitted within this paradigm.

**Supplementary Information:**

The online version contains supplementary material available at (10.1186/s12874-022-01550-8).

## Background

**Fig. 1:**
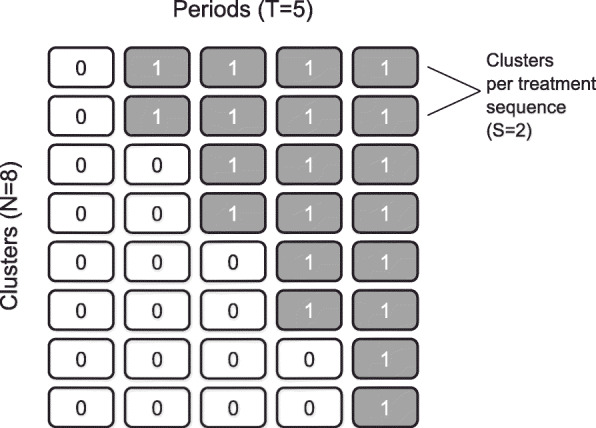
Schematic of a stepped wedge design, with N=8 clusters (rows), T=5 periods (columns), and S=2 clusters per treatment sequence (number of times each unique row is repeated)

Stepped wedge (SW) trials are cluster randomized trial (CRT) designs where clusters are randomized to sequences of intervention and control conditions. All clusters initially implement the control condition for one or more time periods and then switch to the intervention condition for one or more periods. Collectively, clusters roll over from implementing the control condition to the intervention in a stepped manner over the defined time periods of the trial, until all clusters implement the intervention by the final period; an example is displayed in Fig. 1. As with all CRTs, subjects within a cluster are likely to be more similar to each other than to subjects in other clusters, and it is important to account for this similarity in the statistical model we use for the design and analysis of the trial. This similarity is encoded by the correlation between a pair of subjects’ outcomes belonging to the same cluster and is called the intracluster correlation.

SW designs have become increasingly popular in recent years [1] (there were 330 SW trials registered on clinicaltrials.gov as of August 17, 2021) and may offer a number of advantages over some alternative CRT designs. For example, a SW design tends to have improved power over simpler designs such as the parallel design, when the intracluster correlation or the cluster size is large [2]. The design also allows for all clusters to receive the intervention by the end of the trial (the most common reason for use in a 2017 review of 123 planned or completed SW trials) [1] and offers a pragmatic option for settings in which intervention rollout is necessarily extended over time [3] (the most common reason in a 2015 review of 37 SW trials) [4].

SW CRTs often recruit relatively small numbers of clusters: a review of 102 SW trials found that the median number of clusters was 12, with 45% having fewer than 10 clusters [5]. Warnings abound in the CRT literature about the validity of sample size calculations and subsequent data analysis using standard methods for trials with a “small” number of clusters [3, 5, 6]. However, there is little consensus as to what constitutes “small.” Standard methods of analysis such as linear mixed models (LMMs) with estimation via maximum likelihood (ML) or restricted maximum likelihood (REML) rely on their large-sample, asymptotic properties for inference about intervention effects. Use of these methods in small-sample settings, although commonplace [5], has been shown under some scenarios to yield underestimated standard errors for fixed effects such as the intervention effect, resulting in inflated type I error rates [7, 8].

REML with the addition of a small-sample correction has shown promise. For example, REML with a Kenward-Roger (KR) approximation has been shown to help maintain the type I error rate for parallel CRTs with small numbers of clusters [9–11]. And a study on type I error control when testing the intervention effect in parallel CRTs found that a likelihood ratio test with ML gave inflated type I error rates under many scenarios while a Wald test with REML and additional degrees of freedom corrections performed better [12]. The KR approximation is a small-sample correction that produces adjusted standard errors for fixed effects in LMMs and adjusted denominator degrees of freedom for regression coefficients [13, 14]. The adjusted standard errors are obtained using an approximate small sample estimator for the covariance matrix for the fixed effects that better accounts for the uncertainty in estimation of the random effects variance components. This adjustment is widely implemented in software and has become a common approach for computation of standard errors of regression parameters in multilevel models in small-sample contexts. A potentially large limitation of this approach in a CRT context, however, is that it does not provide any adjustment to inference about the variance components and therefore the estimated intracluster correlation, a function of the variance components [15].

An alternative approach to estimation can be implemented using Bayesian methods, although this has seen relatively little uptake so far for CRTs. It was first described in the context of CRTs in 2001 by Spiegelhalter [16] and Turner et al. [17] for continuous and binary outcomes, respectively. However, in a recent review, Jones et al. [18] identified only 11 papers reporting the use of Bayesian methods to analyze parallel CRTs to the year 2018, with severe deficiencies in their reporting and justification. We are aware of only one SW trial analyzed with a Bayesian method [19], and one simulation study including Bayesian estimation in the context of SW trials which focused on the impact of using weakly informative priors for time effects on sample size calculations [20].

Bayesian inference uses probability distributions to characterize (directly) the uncertainty about unknown parameters [21]. We assign the parameters a (joint) prior distribution, which represents the assumptions we are willing to make about the magnitude of the parameters, and then specify the model for the data given the parameters, as for a frequentist analysis, in the form of a likelihood function. The product of the prior density and likelihood function then yields the joint posterior density, up to a normalizing constant. Direct calculation is impossible with realistic models so we obtain inference about the parameters of interest by using an approximate method such as sampling from the joint posterior distribution using Markov Chain Monte Carlo (MCMC) which yields sets of draws from the marginal posterior distributions of the parameters. These sets of draws represent the likely range of values for each parameter of interest, conditioning on the observed data. The inference is richer than what can be obtained from frequentist approaches such as REML estimation, which yields a point estimate for each parameter and estimated standard errors and interval estimates based on the repeated-sampling properties of the estimators.

The choice of prior distributions for a given analysis can be very important and warrants careful consideration, particularly for hierarchical/multilevel models with little replication at higher levels, such as few clusters in SW CRTs [21, 22]. Most Bayesian multilevel models specify diffuse prior distributions for the random effects variance components [23]. However, models for CRTs must account for the similarity between subjects in a cluster through one or more intracluster correlation components, which are simply functions of the variance components, and CRTs now commonly report these estimates [24, 25]. We can therefore typically obtain a plausible range of values for the intracluster correlation parameters, either based on related studies [26] or from past experience. For CRTs with a small number of clusters in particular, there will be little information in the data to inform the intracluster correlation parameter estimates and so utilizing weakly informative priors can assist in estimation. Diffuse priors may be more appropriate for the other parameters in the model such as the treatment effect and time effects. We may prefer not to impose any assumptions about their magnitude, and we expect the data to contain sufficient information to inform their estimation.

In this paper we describe an approach for Bayesian estimation of the treatment effect and intracluster correlation parameters in LMMs appropriate for SW designs, and use a simulation study to compare their repeated-sampling performance with the commonly used REML estimators, with and without the KR correction. In the next section we will set out the simulation study framework and describe the estimation methods and implementation details. Then we will present the simulation study results and provide some concluding remarks.

## Methods

### Simulation study framework

#### Simulation study aim

Our primary aim was to assess the frequentist (i.e., repeated-sampling) properties of estimators for the treatment effect and the intracluster correlation parameters for two approaches to analyzing SW CRTs when the number of clusters is small. Both approaches use an LMM but differ in their method of estimation. One is a frequentist approach using REML estimation with and without a KR small-sample correction, and the other is a Bayesian approach using MCMC estimation with weakly informative prior distributions for the intracluster correlation parameters.

#### Trial design

We considered standard SW trial designs, where all periods are of equal duration, the first and last periods involve only the control and intervention, respectively, and where an equal number of clusters are allocated to each treatment sequence. Then for a trial with *T* periods there are *T*−1 unique treatment sequences and we take the number of clusters *N* to be *N*=*S*(*T*−1) where *S* is the number of clusters assigned to each treatment sequence. We also assume that an equal number of new subjects are recruited at each time period and each subject is measured just once.

#### Linear mixed model: block-exchangeable correlation structure

We let *Y*_*ijk*_ be the continuous measured outcome of subject *k* (*k*=1,…,*m*) measured in period *j* (*j*=1,…,*T*) and belonging to cluster *i* (*i*=1,…,*N*):

1 $$ \begin{aligned} Y_{ijk} &= \mu_{ij} + e_{ijk}, \quad e_{ijk} \sim N\left(0,\sigma_{e}^{2}\right) \\ \mu_{ij} &= \beta_{j} + X_{ij}\theta + C_{i} + (CP)_{ij}, \\ & \quad C_{i} \sim N(0,\sigma_{C}^{2}), \quad (CP)_{ij} \sim N\left(0,\sigma_{CP}^{2}\right) \end{aligned}  $$

where *e*_*ijk*_ is the (random) subject-level error, *β*_*j*_ is the categorical time period (fixed) effect for period *j*, *X*_*ij*_ is a treatment indicator (1 if cluster *i* implements the treatment in period *j*, 0 otherwise), *θ* is the treatment (fixed) effect, *C*_*i*_ is the (random) effect for cluster *i*, and (*CP*)_*ij*_ is the (random) effect for cluster-period (*i*,*j*). We assume that *e*_*ijk*_,*C*_*i*_, and (*CP*)_*ij*_ are all mutually independent.

This model induces a constant between-period correlation structure within a cluster, often referred to as the block-exchangeable model, whereby it is assumed that subjects’ outcomes have one of two magnitudes of correlation [27, 28]. Subjects’ outcomes measured in the same cluster and same time period are assumed to have a correlation given by $\rho _{1} = \frac {{\sigma _{C}}^{2} + {\sigma _{\text {CP}}}^{2}}{{\sigma _{C}}^{2} + {\sigma _{\text {CP}}}^{2} + {\sigma _{e}}^{2}}$, which we will refer to as the within-period intracluster correlation, and subjects’ outcomes measured in the same cluster but different time periods are assumed to have a (lower) between-period intracluster correlation given by $\rho _{2} = \rho _{1}r = \frac {{\sigma _{C}}^{2}}{{\sigma _{C}}^{2} + {\sigma _{\text {CP}}}^{2} + {\sigma _{e}}^{2}}$, where we will refer to $r = \frac {{\sigma _{C}}^{2}}{{\sigma _{C}}^{2} + {\sigma _{\text {CP}}}^{2}}$ as the cluster autocorrelation.

#### Linear mixed model: exchangeable correlation structure

When the cluster autocorrelation *r*=1, model (1) reduces to the exchangeable correlation model [3]:

2 $$ \begin{aligned} Y_{ijk} &= \mu_{ij} + e_{ijk}, \quad e_{ijk} \sim N\left(0,\sigma_{e}^{2}\right) \\ \mu_{ij} &= \beta_{j} + X_{ij}\theta + C_{i}, \quad C_{i} \sim N\left(0,\sigma_{C}^{2}\right) \end{aligned}  $$

The model no longer includes a cluster-period effect and there is just one correlation component, the intracluster correlation, $\rho _{1} = \frac {{\sigma _{C}}^{2}}{{\sigma _{C}}^{2} + {\sigma _{e}}^{2}}$.

#### Generating trial data

We generated trial data for SW designs with *S*=1, 2 and 5 clusters per treatment sequence, *T*=5 and 9 periods and *m*=10 and 100 subjects measured in each cluster-period. The SW designs we considered had *T*−1 unique treatment sequences with number of clusters *N*=*S*(*T*−1), as in Fig. 1. The range of designs with *N* clusters and *T* periods was then (*N*,*T*)∈{(4,5),(8,5),(20,5),(8,9),(16,9),(40,9)}. This yielded cluster sizes ranging from 50 to 900 and a total number of subjects of between 200 and 36,000. We chose ranges of trial design parameters that were broadly aligned with several reviews that reported summary measures of design characteristics for completed and planned SW trials. These reviews reported an interquartile range (IQR) for number of clusters randomized per sequence of 1 to 8 with a median of 3 clusters [29], median numbers of steps (periods - 1) of 4 steps [5, 29] and 9 steps [1], and total numbers of clusters as low as 2 clusters [1, 5] and 4 clusters [4] with a median ranging from 12 clusters [5] to 20.5 clusters [1]. While cluster size and total number of subjects were not as frequently reported, Martin et al. [29] reported an IQR for cluster size of 24 to 326 and Grayling et al. [1] reported total numbers of subjects from completed studies ranging from 123 to 26,456.

In our data generating model, we included a linear time period effect with *β*_*j*_=*j*/*T*,*j*=1,…,*T* and held the subject error variance fixed at $\sigma _{e}^{2}=1$. We set the true value of the treatment effect to *θ*=0, consistent with a null hypothesis of no treatment effect in a frequentist framework. Then for each of the considered designs, we simulated data under models (1) and (2); that is, under two within-cluster correlation structures. For each, two within-period intracluster correlations were used: 0.05 and 0.1. For data generated under the block-exchangeable model (model (1)), we simulated data using a cluster autocorrelation of 0.8. Such correlation values largely align with those typically seen in longitudinal CRTs [30]. Table 1 gives the range of values for each trial configuration parameter and correlation parameter that we varied in the simulation study.

**Table 1:** Range of trial configuration and correlation parameter values varied in simulation study

Parameter	Meaning	Values
*S*	Number of clusters per sequence	1, 2, 5
*T*	Number of periods	5, 9
*m*	Number of subjects per cluster-period	10, 100
*ρ*_1_	Within-period intracluster correlation	0.05, 0.1
*r*	Cluster autocorrelation	0.8, 1

Note that we only considered analyses based on correctly specified models in this simulation study; that is, we fit model (1) with the block-exchangeable correlation structure to the datasets arising from configurations with *r*=0.8 and we fit model (2) with the exchangeable correlation structure to datasets where *r*=1 (and we therefore did not estimate *r* for these configurations). Analyses involving model misspecification, while valuable, are beyond the scope of this paper.

#### Estimands

We were primarily interested in inference for the treatment effect, *θ*, as this is the target of a trial analysis, and the intracluster correlation parameters, *ρ*_1_ and *r* (for configurations where *r*≠1), which can be used to inform sample size and power calculations for future trials.

### Estimation methods and implementation

#### Frequentist REML estimation

We used the lme4 [31] (v1.1-26) package in R [32] 4.0.0 to fit the LMM with estimation via REML. For all but the largest configuration, we also applied the KR approximation with the pbkrtest [33] (v0.5-0.1) package to obtain adjusted standard errors and adjusted degrees of freedom for the treatment effect. We used these adjusted values to construct adjusted 95% confidence intervals for the treatment effect, taking the test statistic to be the 97.5^th^ percentile of a *t* distribution with the adjusted degrees of freedom. Note that the KR approximation for the largest configuration (*T*=9,*m*=100,*S*=5) frequently failed due to large memory requirements; however, since the configuration involved a relatively large number of clusters (40), for all replicates of this configuration we instead obtained the equivalent adjusted degrees of freedom using the Satterthwaite approximation [34] with the parameters [35] (v0.11.0) and lmerTest [36] (v3.1-3) packages and retained the unadjusted standard error.

#### Bayesian MCMC estimation

**Fig. 2:**
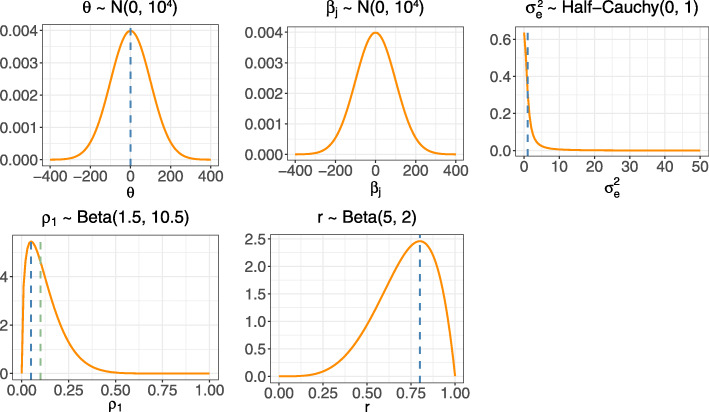
Prior distributions for the Bayesian method. Dashed vertical lines indicate the location of the true parameter values chosen for the simulation study (note that the true values for *β*_*j*_,*j*=1,…,*T* depend on the number of periods and so are not shown here)

For the Bayesian approach, we specified diffuse prior distributions for the treatment effect, period effects, and subject-level error variance. We do not tend to have much prior information about these parameters, and we expect that the data will provide adequate information for their estimation. We specified normal prior distributions of *N*(0,10^4^) for the treatment effect, *θ*, and period effects, *β*_*j*_, and a half-Cauchy(0, 1) prior for the subject-level error variance, $\sigma _{e}^{2}$. The half-Cauchy prior has been recommended for variance components in hierarchical models, though more attention has been paid to the cluster variance than to the error variance [10, 37]. The intracluster correlation parameters can take values in the range 0 to 1, and CRTs in health settings tend to see estimated within-period intracluster correlations ranging from 0.01 to 0.2 with values around 0.05 being most common [30]. We therefore selected a Beta(1.5,10.5) prior distribution which has a mode of 0.05 and is right-skewed with low probability mass for values greater than 0.2. CRTs that assume a block-exchangeable correlation structure as in model (1) tend to estimate a cluster autocorrelation of between 0.5 and 1, with values in the upper end of this range being more likely [30, 38]. A Beta(5,2) prior captured this range of values well, being left-skewed over smaller values and having the most probability mass around 0.8. We considered these prior distributions for the intracluster correlation parameters to be weakly informative, as they were intended to be applicable to CRTs more generally and were not tailored to a particular trial or outcome measure. Figure 2 shows each of these prior distributions along with the parameter values used to simulate the data.

Note that the prior distributions for the within-period intracluster correlation, *ρ*_1_, cluster autocorrelation, *r*, and error variance, $\sigma _{e}^{2}$, imply distributions for the cluster variance, $\sigma _{C}^{2}$, and cluster-period variance, $\sigma _{CP}^{2}$ (see Section A in Additional file 1 for the associated formulae and plots of these distributions). Also note that for trial configurations where *r*=1, we do not estimate *r* as we are essentially specifying a very strong prior distribution (where *P*(*r*=1)=1); we specified the same Beta(1.5,10.5) prior for *ρ*_1_ and the same prior distributions for the reduced set of parameters as above.

We used Stan in R via the rstan [39] (v2.21.2) package to fit the LMM using a Bayesian approach with estimation via MCMC. The MCMC algorithm was the no-U-turn sampler (NUTS), a variant of Hamiltonian Monte Carlo, and we set the target average acceptance probability (the adapt_delta parameter in Stan) to 0.95 (increased from the default of 0.8 to yield a smaller step size during sampling with an aim to reducing the occurrence of divergent transitions after warmup) [40]. For each model fit, we ran four chains, each for 1000 warmup iterations followed by 5000 post-warmup iterations. We found this to be a sufficient number of iterations in pilot analyses to yield adequate diagnostics.

We assessed convergence with the potential scale reduction factor split-$\widehat {R}$, concluding adequate mixing of chains if split-$\widehat {R} < 1.01$ for all parameters [41]. We also included checks that the rank-normalized Bulk Effective Sample Size (ESS) and Tail ESS for all parameters exceeded a lower threshold of 400 to ensure stable estimates of the uncertainty in the marginal posterior distributions.

#### Performance measures

To assess method performance we computed bias and mean squared error (MSE) for the treatment effect and correlation parameters, as well as the coverage of 95% confidence/credible intervals and relative percent error in model-based standard error for the treatment effect. Note that we could not obtain 95% confidence intervals or relative percent error in model-based standard error for the correlation parameters as the REML method in lme4 does not produce standard errors or confidence intervals for the variance components. As the KR approximation does not affect the treatment effect estimate, bias and MSE for the treatment effect are shown just for REML and the Bayesian approach, while interval coverage and relative percent error in model-based standard error are shown for REML, REML (KR), and the Bayesian method. For the Bayesian method, we summarized the marginal posterior distribution for each parameter with the posterior median, which was then used for calculating the bias and MSE. While we expected the marginal posterior distributions for the treatment effect to be fairly symmetric, we supposed that those for the correlation parameters would likely be skewed given the boundaries at 0 and 1 and therefore chose to summarize the distributions with the median rather than the mean which would have been more sensitive to skewness. We used the 2.5^th^ and 97.5^th^ percentiles of the marginal posterior distributions to define the 95% credible interval.

For each combination of trial configuration parameters we generated *n*_sim_=1000 datasets and applied both estimation methods to each dataset. Our choice of *n*_sim_ was largely motivated by computation time, as both the MCMC sampling and the KR approximation could be time-consuming for larger configurations and we were subject to maximum computation time constraints on the computing cluster we used. We also calculated the associated Monte Carlo standard errors (MCSEs) for each performance measure to estimate simulation uncertainty, which we include in the results tables in Section B in Additional file 1. Table 2 gives the definitions and expressions for the performance measures and associated MCSEs, calculated according to Morris et al. [42]. Note that we would expect the MCSE associated with 95% interval coverage to be ± 0.7*%*.

**Table 2:** Definitions and expressions for calculating performance measure estimates and associated Monte Carlo standard errors (MCSEs)

Performance Measure	Definition	Estimate ^*a*^	MCSE of Estimate
Bias	$\mathrm {E}[\hat {\theta }] - \theta $	$\frac {1}{n_{\text {sim}}} \sum _{i=1}^{n_{\text {sim}}} \hat {\theta }_{i} - \theta $	$\sqrt {\frac {1}{n_{\text {sim}}(n_{\text {sim}}-1)} \sum _{i=1}^{n_{\text {sim}}} (\hat {\theta }_{i} - \bar {\theta })^{2}}$
MSE	$\mathrm {E}[(\hat {\theta } - \theta)^{2}]$	$\frac {1}{n_{\text {sim}}} \sum _{i=1}^{n_{\text {sim}}} (\hat {\theta }_{i} - \theta)^{2}$	$\sqrt {\frac {\sum _{i=1}^{n_{\text {sim}}} \left [ (\hat {\theta }_{i} - \theta)^{2} - \widehat {\text {MSE}} \right ]^{2}}{n_{\text {sim}}(n_{\text {sim}} - 1)}}$
Coverage	P$(\hat {\theta }_{\text {low}} \le \theta \le \hat {\theta }_{\text {upp}})$	$\frac {1}{n_{\text {sim}}} \sum _{i=1}^{n_{\text {sim}}} 1 (\hat {\theta }_{\text {low},i} \le \theta \le \hat {\theta }_{\text {upp},i})$	$\sqrt {\frac {\widehat {\text {Cover.}} \times (1 - \widehat {\text {Cover.}})}{n_{\text {sim}}}}$
Average ModSE ^*b*,*c*^	$\sqrt {\mathrm {E}[\widehat {\text {Var}}(\hat {\theta })]}$	$\sqrt {\frac {1}{n_{\text {sim}}} \sum _{i=1}^{n_{\text {sim}}} \widehat {\text {Var}}(\hat {\theta }_{i})}$	$\sqrt {\frac {\widehat {\text {Var}}[\widehat {\text {Var}}(\hat {\theta })]}{4 n_{\text {sim}} \times \widehat {\text {ModSE}}^{2}}}^{d}$
EmpSE ^*e*^	$\sqrt {\text {Var}(\hat {\theta })}$	$\sqrt {\frac {1}{n_{\text {sim}}-1} \sum _{i=1}^{n_{\text {sim}}} (\hat {\theta }_{i} - \bar {\theta })^{2}}$	$\frac {\widehat {\text {EmpSE}}}{\sqrt {2 (n_{\text {sim}} - 1)}}$
Relative % error in ModSE ^*b*,*c*^	$100 \left (\frac {\text {ModSE}}{\text {EmpSE}} - 1 \right)$	$100 \left (\frac {\widehat {\text {ModSE}}}{\widehat {\text {EmpSE}}} - 1 \right)$	$100 \left (\frac {\widehat {\text {ModSE}}}{\widehat {\text {EmpSE}}} \right) \sqrt {\frac {\widehat {\text {Var}}[\widehat {\text {Var}}(\hat {\theta })]}{4 n_{\text {sim}} \times \widehat {\text {ModSE}}^{4}} + \frac {1}{2(n-1)}}^{d}$

Source: Morris et al. [42]

^a^ *θ* is the parameter of interest, $\hat {\theta }_{i}$ is the parameter estimate for replicate *i*, $\bar {\theta }$ is the mean estimate across all replicates, and *n*_sim_ is the total number of replicates

^b^ModSE is the model-based standard error

^c^MCSEs are approximate for Average ModSE and Relative % error in ModSE

^d^$\widehat {\text {Var}}[\widehat {\text {Var}}(\hat {\theta })] = \frac {1}{n_{\text {sim}} - 1} \sum _{i=1}^{n_{\text {sim}}} \{ \widehat {\text {Var}}(\hat {\theta }_{i}) - \frac {1}{n_{\text {sim}}} \sum _{j=1}^{n_{\text {sim}}} \widehat {\text {Var}}(\hat {\theta }_{j}) \}^{2}$

^e^EmpSE is the empirical standard error

#### Computation

We ran the simulation study on the Monash MASSIVE high performance computing cluster [43]. To reduce total computation time, we parallelized computation across the 48 trial configurations as well as across batches of replicates for some of the larger and more time-intensive configurations for a total of 156 jobs, using one CPU core per job. We used R version 4.0.0, lme4 version 1.1-26 and rstan version 2.21.2. For session information and package dependencies for lme4 and rstan, see Section C in Additional file 1. Project source code is available at https://github.com/klgrantham/bayesian-SW.

## Results

### Performance measures for the treatment effect

**Fig. 3:**
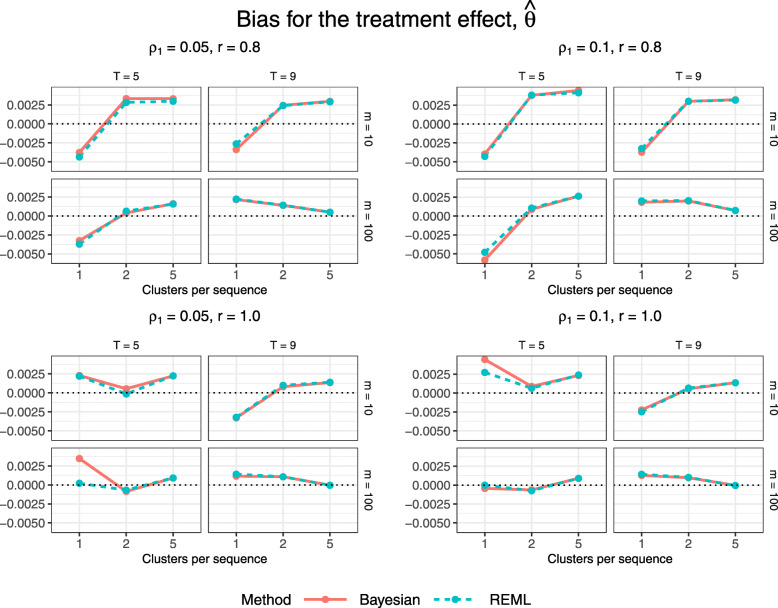
Estimated bias for $\hat {\theta }$ across all trial configurations. See also Table B1, Additional file 1

**Fig. 4:**
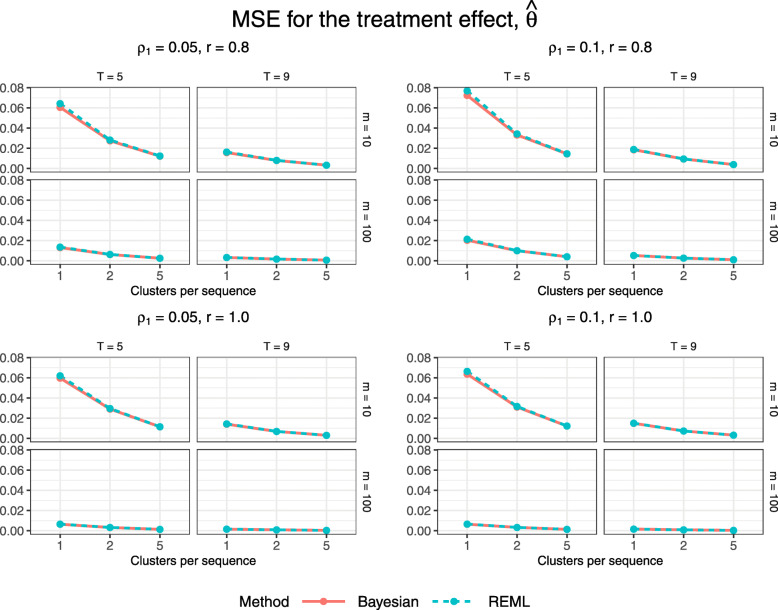
Estimated MSE for $\hat {\theta }$ across all trial configurations. See also Table B2, Additional file 1

Figure 3 displays estimated bias for the treatment effect estimator, $\hat \theta $, across all trial configurations. Each quadrant corresponds to a different combination of true correlation parameter values, each of the four plots within a quadrant corresponds to a different combination of number of periods and subjects per cluster-period, and the dots within each plot correspond to a different value of *S*∈{1,2,5}, the number of clusters per unique treatment sequence (recall that configurations with *T*=5 periods have *N*=*S*(*T*−1)∈{4,8,20} clusters, and those with *T*=9 have *N*∈{8,16,40} clusters). Both the Bayesian and REML methods yield similar bias with the exception of slight differences for the smallest configurations with one or two clusters per sequence, for which neither method consistently outperforms the other. Figure 4 displays estimated MSE for $\hat \theta $. Both estimation methods yield nearly identical MSE. The Bayesian method appears to yield slightly lower MSE for the smallest configurations with just one cluster per treatment sequence and the smaller cluster-period size of 10, however this could simply reflect simulation uncertainty as the values are only about one MCSE apart (Table B2, Additional file 1). The magnitude of the MSE for both methods decreases with the inclusion of more clusters per sequence and therefore more total subjects in the trial.

**Fig. 5:**
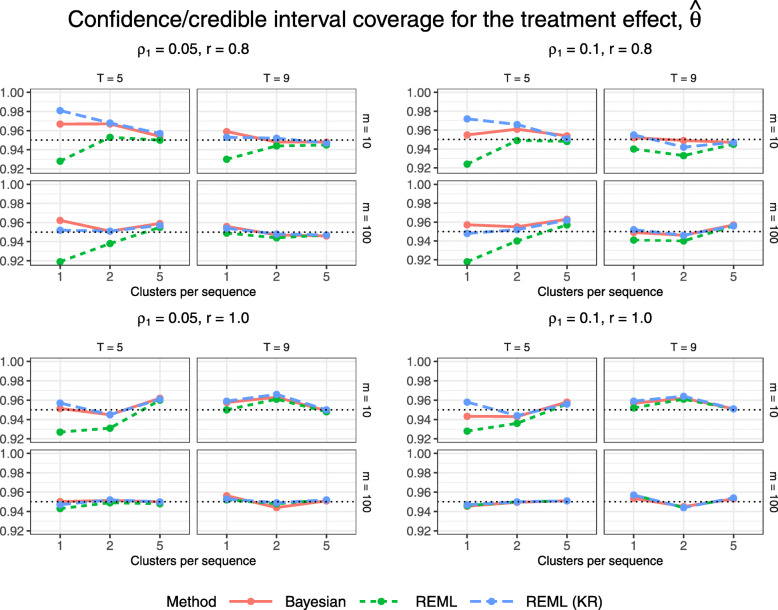
Estimated confidence/credible interval coverage for $\hat {\theta }$ across all trial configurations. See also Table B3, Additional file 1

**Fig. 6:**
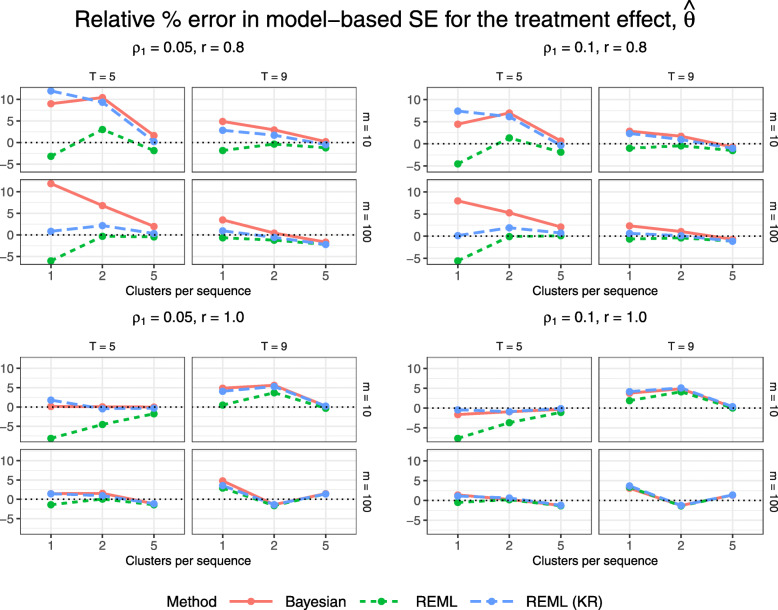
Estimated relative percent error in model-based standard error for $\hat {\theta }$ across all trial configurations. See also Table B4, Additional file 1

**Fig. 7:**
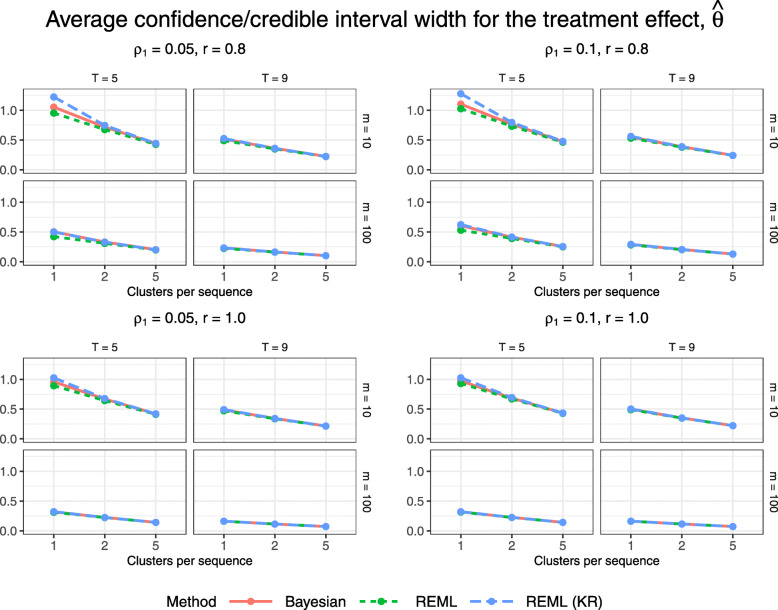
Widths of estimated confidence/credible intervals for $\hat {\theta }$ across all trial configurations. See also Table B5, Additional file 1

Figures 5 and 6 display 95% confidence/credible interval coverage and the relative percent error in model-based standard error, for the Bayesian method and for REML with and without the KR approximation. The Bayesian method and REML with the KR approximation achieve coverage close to 95% for all configurations; the Bayesian method is the only method to achieve coverage within 1.4% (within 2*MCSE) of the nominal 95% level, while REML with the KR approximation is overly conservative for some of the smallest configurations with just one cluster per sequence, 5 periods, and 10 subjects per cluster-period. REML estimation without the KR approximation does not tend to achieve 95% coverage for configurations with few clusters (1 or 2 clusters per sequence). This low coverage would be consistent with an inflated type I error rate were we to conduct a hypothesis test for a non-null treatment effect, as the trial data were generated under a null treatment effect. We see a similar relationship between the methods in Fig. 6 as in Fig. 5, where having a higher relative percent error in model-based standard error tends to correspond to a higher interval coverage. Confidence/credible interval widths vary slightly across methods for configurations with just one cluster per sequence, but neither the Bayesian method nor REML with the KR approximation gives clearly narrower intervals (Fig. 7).

### Performance measures for the correlation parameters

**Fig. 8:**
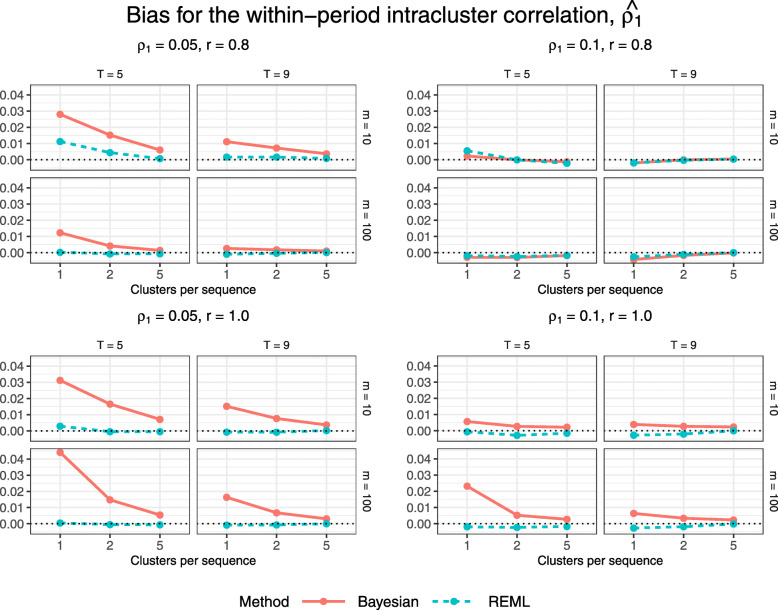
Estimated bias for $\widehat {\rho _{1}}$ across all trial configurations. See also Table B6, Additional file 1

**Fig. 9:**
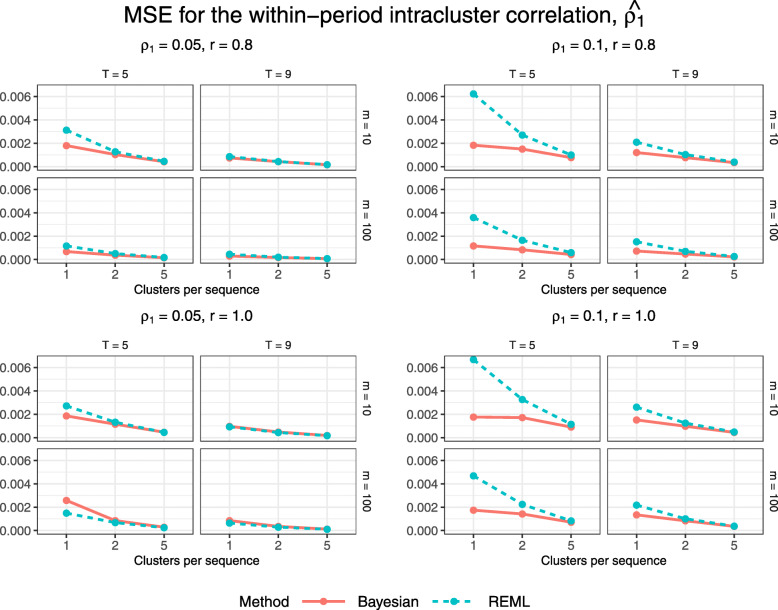
Estimated MSE for $\widehat {\rho _{1}}$ across all trial configurations. See also Table B7, Additional file 1

Figures 8 and 9 display estimated bias and MSE for the estimated within-period intracluster correlation, $\widehat {\rho _{1}}$. The Bayesian method gives higher bias but lower MSE than REML estimation for most configurations. For each combination of true correlation parameter values and numbers of periods and subjects per cluster-period, absolute bias and MSE decrease toward zero with increasing numbers of clusters per treatment sequence.

**Fig. 10:**
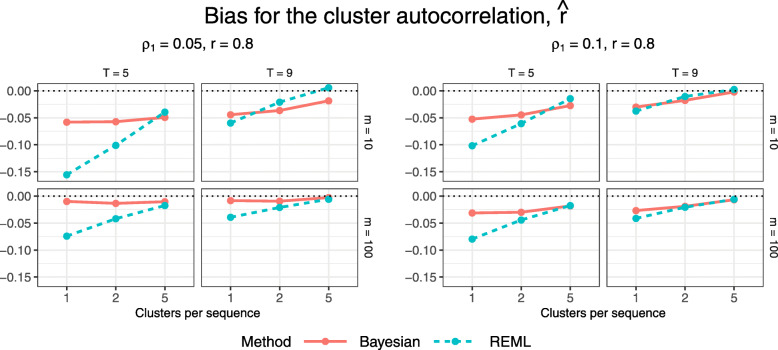
Estimated bias for $\hat {r}$ across all trial configurations. See also Table B8, Additional file 1

**Fig. 11:**
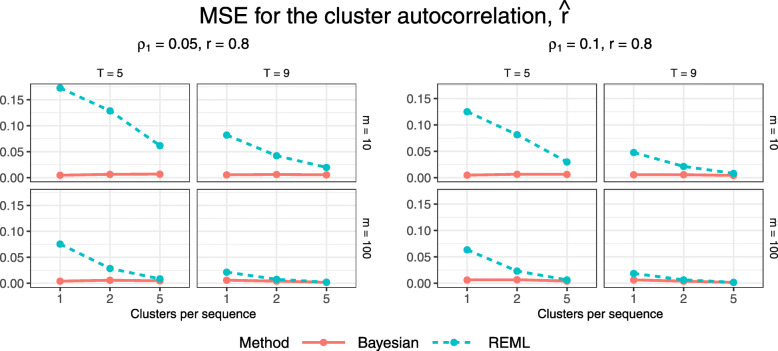
Estimated MSE for $\hat {r}$ across all trial configurations. See also Table B9, Additional file 1

Figures 10 and 11 display estimated bias and MSE for the estimated cluster autocorrelation, $\hat {r}$, for configurations with an underlying block-exchangeable correlation structure (*r*=0.8). Absolute bias tends to be greater for REML than for the Bayesian method for smaller configurations but the two methods yield similar bias for larger configurations. The estimation methods differ more markedly in their performance according to MSE. REML gives higher MSE for nearly all configurations except the largest few, for which the MSE under both methods is near zero, while the Bayesian method gives very low MSE for all configurations. The observed high MSE under REML can likely be attributed, at least in part, to poor estimation of the variance components for some simulation replicates: we found that REML estimation may incorrectly estimate one or both of the cluster and cluster-period variances as zero. Since $\hat {r}=\frac {\widehat {\sigma _{C}^{2}}}{\widehat {\sigma _{C}^{2}} + \widehat {\sigma _{CP}^{2}}}$, this would result in extreme estimates of $\hat {r}=0$ and 1 when $\widehat {\sigma _{C}^{2}}=0$ and $\widehat {\sigma _{CP}^{2}}=0$, respectively. Note that we excluded any replicates for which both of these variances were estimated as 0 as this would yield an invalid estimate of *r*; we provide more detail in the next subsection.

### Invalid simulation replicates

Some simulation replicates yielded invalid results and were therefore excluded from calculation of the particular method’s performance measures. For the Bayesian method, we dropped replicates which yielded any divergent transitions or failed to meet the diagnostic criteria described previously in the Estimation methods and implementation subsection. In Stan, divergent transitions during MCMC sampling are indicative of a rough optimization surface which the algorithm struggled to navigate, suggesting that inference from a model fit which yields divergent transitions may be invalid [40]. For REML estimation, we dropped replicates from the calculation of performance measures for the cluster autocorrelation which estimated both the cluster and the cluster-period variances as zero, as this gives an invalid estimate of the cluster autocorrelation. We retained all replicates for the calculation of performance measures for the other parameters. Table 3 gives the percentage of retained replicates for each trial configuration by method, with the REML columns showing the percentage of retained replicates for the calculations pertaining to the cluster autocorrelation. No replicates were excluded for any of the configurations with *S*=5 clusters per sequence, for either method. Small proportions of replicates were invalid for both methods for configurations with *r*=0.8 and *S*=1 cluster per sequence, and REML estimation also yielded a smaller proportion of invalid replicates for some configurations with *S*=2. For configurations with *r*=1.0, note that REML estimation could not yield invalid estimates under our definition of invalid as the cluster-period term, and therefore the cluster autocorrelation, do not appear in the model. MCMC estimation gave moderate proportions of invalid replicates for three of the four configurations with *S*=1 and *m*=100 and small proportions of invalid replicates for some of the remaining configurations with *S*=1 or 2.

**Table 3:** Percentage of valid simulation replicates across *n*_sim_=1000 replicates. Bayesian replicates were excluded if they yielded any divergent transitions, effective sample sizes were too low (below 400), or split-$\hat {r}$ values were too large (above 1.01). REML replicates were excluded from calculations for $\hat {r}$ if both cluster and cluster-period variances were estimated as 0, yielding an invalid estimate of *r*

				*r*			
				0.8		1	
*ρ*_1_	T	m	S	Bayesian	REML	Bayesian	REML
0.05	5	10	1	99.5	88.5	94.6	100.0
			2	100.0	97.4	99.8	100.0
			5	100.0	100.0	100.0	100.0
		100	1	90.1	99.8	44.2	100.0
			2	100.0	100.0	94.6	100.0
			5	100.0	100.0	100.0	100.0
	9	10	1	100.0	99.6	99.1	100.0
			2	100.0	99.9	100.0	100.0
			5	100.0	100.0	100.0	100.0
		100	1	99.8	100.0	86.9	100.0
			2	100.0	100.0	100.0	100.0
			5	100.0	100.0	100.0	100.0
0.1	5	10	1	99.8	95.2	97.0	100.0
			2	100.0	99.9	100.0	100.0
			5	100.0	100.0	100.0	100.0
		100	1	98.0	100.0	67.8	100.0
			2	100.0	100.0	99.0	100.0
			5	100.0	100.0	100.0	100.0
	9	10	1	100.0	100.0	99.6	100.0
			2	100.0	100.0	100.0	100.0
			5	100.0	100.0	100.0	100.0
		100	1	100.0	100.0	96.8	100.0
			2	100.0	100.0	100.0	100.0
			5	100.0	100.0	100.0	100.0

For a brief exploration of results from the configuration with the largest proportion of invalid Bayesian replicates (*S*=1,*T*=5,*m*=100 with *ρ*_1_=0.05 and *r*=1), we compared the parameter estimates from the 442 valid replicates with those from the 558 invalid replicates, noting that the latter set of replicates were all deemed invalid for yielding divergent transitions. While the posterior medians for the treatment effect estimates were similar between the two sets of replicates, the posterior medians for the within-period intracluster correlation from the set of invalid replicates tended to be smaller and closer to the boundary at 0 (Section D, Additional file 1). Based on this clear difference in the magnitude of estimates, we can speculate that many of these divergent transitions occurred when the algorithm had difficulty exploring very small correlation parameter values, as has also been shown to occur for the group variance parameter in similar hierarchical models [44].

### Illustrative example

**Fig. 12:**
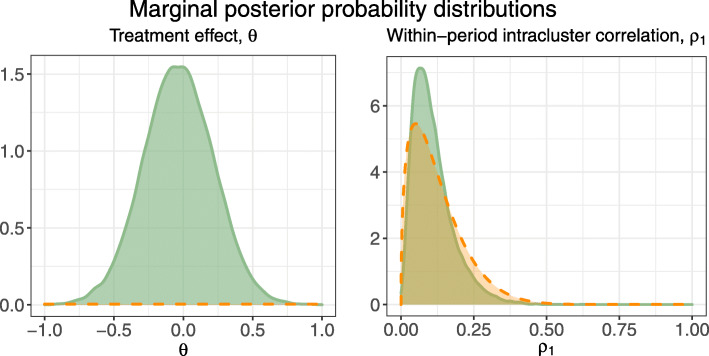
Posterior distributions (solid lines) for the treatment effect and within-period intracluster correlation, obtained from the analysis of a randomly-selected simulated dataset for a SW design with *S*=1,*T*=5,*m*=10, true treatment effect *θ*=0, and intracluster correlation parameters *ρ*_1_=0.1, and *r*=1. Prior distributions (dashed lines) are overlaid for reference. Note that the prior for the treatment effect is *N*(0,10^4^) with a probability density of 0.004 at *θ*=0

While the primary aim of this paper was to evaluate the repeated-sampling performance of the methods, we also give an example of the inference obtained from the methods for a single simulated dataset. We randomly selected one simulated dataset from the trial configuration with *S*=1,*T*=5,*m*=10,*ρ*_1_=0.1, under the exchangeable correlation model (*r*=1). Table 4 displays point estimates and 95% confidence/credible intervals for the treatment effect, *θ*, and the within-period intracluster correlation, *ρ*_1_, for REML with the KR approximation, REML without the KR approximation, and the Bayesian method, where the same prior distributions and implementation settings were used as in the simulation study. For ease of comparison across methods, we have summarized the posterior probability distributions from the Bayesian method by the medians and 95% credible intervals. The marginal posterior distributions for these parameters are shown in full in Fig. 12 with the prior distributions overlaid for reference.

**Table 4:** Inference for the treatment effect, *θ*, and the within-period intracluster correlation, *ρ*_1_, for a randomly-selected simulated dataset for a SW design with *S*=1,*T*=5,*m*=10, true treatment effect *θ*=0, and intracluster correlation parameters *ρ*_1_=0.1 and *r*=1. Estimate and 95% CI correspond to point estimates and 95% confidence intervals for the REML methods and medians of posterior draws and 95% credible intervals for the Bayesian method. Note that standard errors for *ρ*_1_ are not provided in lme4 to permit 95% confidence intervals for the REML methods

	*θ*		*ρ*_1_	
Method	Estimate	95% CI	Estimate	95% CI
REML (KR)	-0.033	(-0.568, 0.503)	0.071	-
REML	-0.033	(-0.525, 0.460)	0.071	-
Bayesian	-0.034	(-0.533, 0.463)	0.094	(0.020, 0.285)

Had we analyzed this dataset using any of these methods, we would have drawn very similar conclusions about the treatment effect but obtained slightly different inference for the within-period intracluster correlation. The point estimate for the treatment effect obtained using REML is virtually identical to the median of the Bayesian marginal posterior distribution. All three 95% intervals include 0, the treatment effect value used in generating the dataset; REML with the KR approximation gives the widest interval, while the Bayesian credible interval is slightly wider than REML without the KR approximation. Note also in Fig. 12 that the marginal posterior distribution for the treatment effect is much more sharply peaked around zero than the diffuse *N*(0,10^4^) prior distribution we specified. For the within-period intracluster correlation, REML gives an estimate of $\hat {\rho _{1}}=0.071$ while the median of the Bayesian marginal posterior distribution for *ρ*_1_ is 0.094, closer to the true value of 0.1. Richer inference is possible with the Bayesian method than with REML: the marginal posterior distribution for the treatment effect could be used to address research questions such as the probability that the treatment effect is greater than a particular value, while the marginal posterior distribution for the within-period intracluster correlation could better inform the sample size and power calculations for future related trials than a point estimate alone as can be obtained from REML.

## Discussion

In this paper we have performed a simulation study of the repeated-sampling properties of Bayesian estimation in SW trials, and compared their performance to those of REML estimators from LMMs. We found that both estimation methods provided similar inference for the treatment effect but differed in their ability to estimate the intracluster correlation parameters. For estimation of the treatment effect: there was little bias for both methods, even for a small number of clusters, and the MSEs were virtually identical; and credible interval coverage was appropriate for the Bayesian method and for confidence interval coverage for REML with the KR adjustment, with both methods having similar interval widths. For estimation of the within-period intracluster correlation, we found slightly greater bias for the Bayesian method but far lower MSE. However, for the cluster autocorrelation, the Bayesian method had lower bias and MSE than REML estimation for most configurations. Differences in performance were more pronounced for configurations with smaller numbers of clusters and a smaller cluster-period size, and tended to reduce toward very similar performance for the configurations with larger numbers of clusters and a larger cluster-period size.

Our results for REML estimation are consistent with several studies for parallel CRTs where a KR approximation or similar degrees of freedom correction were found to better maintain the type I error rate [9, 11, 12]. Nugent and Kleinman [12] found adequate control of the type I error rate for intervention effect estimates when the Wald test was used with a *t* distribution with a degrees of freedom correction (either between-within or Satterthwaite) which accords with our adequate 95% confidence interval coverage with REML estimation and a KR correction. Our findings are also fairly consistent with several other studies comparing a Bayesian approach with MCMC estimation to a frequentist approach with REML estimation for multilevel and related models: similar performance in estimating regression coefficients such as the treatment effect, but differential performance for variance components [10, 15, 23]. Specifically, the studies that included a measure of variability of the estimates found that a Bayesian method tended to yield lower variability (e.g. MSE, root MSE) but higher bias than alternative frequentist approaches for variance component estimates. While Baldwin and Fellingham [15] acknowledge and accept this bias-efficiency tradeoff, Smid et al. [23] conclude that the higher bias from a Bayesian approach is a limitation and suggest that thoughtful choices of prior distributions for all parameters are needed to help overcome this drawback. An alternative perspective is that bias should not necessarily be the most important criterion when sample sizes are small and that less variability may be preferable in practice [15, 45]. Indeed, the use of a weakly informative prior is likely to yield some bias and so slightly biased estimates may not necessarily be a sign of poor performance with this approach. McNeish and Stapleton [10] also found that REML and Bayesian methods gave similar bias for the treatment effect (except in the case of four clusters, where REML was more biased) but less similar results for variance component estimates. The bias for the cluster variance from the Bayesian method varied across the three choices of prior distributions and the magnitude of bias from REML fell above and below the Bayesian method for different numbers of clusters and cluster sizes. We note that the McNeish and Stapleton [10] study considered smaller cluster sizes more appropriate for psychological studies and so the greater impact from a small number of clusters that they observed is not surprising.

In our simulation study to compare these methods, we performed a frequentist evaluation of a Bayesian approach, meaning that we employed the notion of repeated sampling and the concept of true parameter values as well as the associated performance measures. We collapsed marginal posterior distributions for the parameters of interest to analogues of frequentist summary measures like the posterior median and 95% credible interval. A fully Bayesian analysis would take advantage of the richer inference available in these marginal posterior distributions and allow us to address more sophisticated research questions (such as the probability that the treatment effect is above a certain value without the need for a dichotomous decision rule as in hypothesis testing) as well as capture more of the uncertainty surrounding the likely range of values for all parameters.

We parameterized the Bayesian model in terms of the intracluster correlation rather than the cluster and cluster-period variances so that we could incorporate our knowledge about the likely range of correlation values into the prior distribution. In our experience, trialists usually have far more information about values of correlation parameters than they do about variance components, largely because correlations are invariant to the scale of the particular outcome measure, unlike variance components. Particularly in situations where the number of clusters is small, the data alone will not provide much information about the parameters, and so it is important to make use of any additional information we may have through weakly informative prior distributions. Had we instead used a more common parameterization in terms of variance components, we would have struggled to find suitable distributions such that the implied correlation assumed realistic values. For example, inverse gamma prior distributions on both variance components is a common choice [10, 23] but implies an unrealistic U-shaped prior distribution for the intracluster correlation with most mass around the extreme values of 0 and 1 [16]. One advantage of this parameterization, however, is that more is known about its behavior: for instance, that inference can be particularly sensitive to the choice of prior distribution for the group variance when the number of groups is small [37]. The parameterization we have used in terms of the intracluster correlation is also likely to be sensitive to the choice of prior distribution. Although we have not formally assessed the sensitivity in this paper, we performed limited simulations specifying a flat prior for the within-period intracluster correlation under the exchangeable correlation model and under this scenario we encountered higher proportions of replicates with divergent transitions. In addition, we observed higher MSE for the Bayesian method than for REML in estimating the within-period intracluster correlation, negating the advantage of the Bayesian method. It is also worth noting that if we are deriving a prior distribution for the intracluster correlation based on reported correlation estimates from past studies with a small number of clusters where inappropriate methods were used or when these estimates were made with great uncertainty, then this derived prior distribution may be problematic.

On a technical note, we found that an alternative coding of the model in Stan, well suited for Bayesian estimation of hierarchical models, helped to improve the sampling efficiency and reduce the occurrence of invalid replicates and we subsequently employed it throughout. Specifically, implementing a non-centered parameterization where hyperparameters are coded as derived quantities rather than drawn from the hyperprior distribution directly as in a centered parameterization yielded lower autocorrelation among parameter draws, higher effective sample sizes, and fewer divergent transitions for most configurations [44]. This parameterization can yield a simpler geometry that allows the algorithm to better explore the range of posterior parameter values, particularly when Hamiltonian Monte Carlo is used [22, 41, 46]. Note that we still encountered divergent transitions after warmup for some configurations but we excluded these replicates from calculation of the performance measures.

Of course, our paper has a number of limitations and more work is needed to establish whether these findings hold with deviations from our particular choices and under a wider range of scenarios. For instance, we considered one set of prior distributions, assumed relatively simple within-cluster correlation structures, and assumed that the analysis models were correctly specified (in particular, that the analysis model assumed the same within-cluster correlation structure that the data was generated with). We also acknowledge that we generated trial data with a treatment effect of zero and specified a prior distribution for the treatment effect with most mass around zero. However, we expected the same or very similar results with non-null treatment effect values. The treatment effect is simply a location shift in the LMM and should not affect REML estimation, and the prior distribution for the treatment effect was so diffuse that it would be unlikely to influence the Bayesian inference. Indeed, from a limited assessment where we generated data with non-null treatment effects, we obtained identical results with REML and very similar results (to within Monte Carlo error) for the Bayesian method (See Section E, Additional file 1). Future work is needed to assess sensitivity to the choice of alternative prior distributions, investigate the implications of more complex within-cluster correlation structures such as discrete-time [47] and continuous-time correlation decay [48], and evaluate method performance when the model is misspecified (for instance, by specifying an overly simplistic within-cluster correlation structure when the correlation structure in the data is more complex, or by assuming an inappropriate form for the time trend). Related work is also needed to assess these methods under other scenarios such as those involving unequal cluster-period sizes, outcome types requiring nonlinear mixed models such as a binary or count outcome, and other CRT designs such as the cluster randomized crossover design.

## Conclusions

Based on the initial investigation in this paper, neither the Bayesian method nor REML with the KR approximation clearly outperformed the other. Rather, they both appear to be viable methods of analysis for SW trials with a continuous outcome and a small number of clusters, with different strengths and weaknesses. The standard REML method together with the KR approximation performed well even with a very small number of clusters in these simulations, although confidence interval coverage was slightly too conservative in some cases. This approach is simpler to implement than a Bayesian analysis, but the inference is not as rich. The Bayesian method incorporates prior information on the parameters, which can aid parameter estimation when the data are limited. However, given the similar performance of the methods, the added complexity of a fully Bayesian analysis may not be warranted, unless the other advantages of Bayesian inference are desired such as the ability to provide probability-based inferences for any parameter of interest.

## Supplementary Information

**Additional file 1** Contains the following sections:
A: Variance component formulae and implied distributions
B: Results tables of performance measures and associated Monte Carlo standard errors
C: Session info and package dependencies
D: Exploratory analysis comparing valid with invalid Bayesian replicates
E: Exploratory analysis comparing results with different treatment effect values

## Data Availability

The datasets analyzed in the simulation study were generated from code available in the GitHub repository: https://github.com/klgrantham/bayesian-SW.

## Abbreviations

SW: Stepped wedge
CRT: Cluster randomized trial
LMM: Linear mixed model
ML: Maximum likelihood
REML: Restricted maximum likelihood
KR: Kenward-Roger
MCMC: Markov Chain Monte Carlo
IQR: Interquartile range
NUTS: No-U-turn sampler
ESS: Effective sample size
MSE: Mean squared error
SE: Standard error
MCSE: Monte Carlo standard error
