# Supplementary material for: Evaluating the performance of Bayesian and restricted maximum likelihood estimation for stepped wedge cluster randomized trials with a small number of clusters
Source: BMC Med Res Methodol. 2022 Apr 13;22:112. doi: 10.1186/s12874-022-01550-8 (PMC9009029; doi:10.1186/s12874-022-01550-8)
Supplement: Supplementary file 1 — Additional file 1 Contains the following sections: A: Variance component formulae and implied distributions B: Results tables of performance measures and associated Monte Carlo standard errors C: Session info and package dependencies D: Exploratory analysis comparing valid with invalid Bayesian replicates E: Exploratory analysis comparing results with different treatment effect values [file 12874_2022_1550_MOESM1_ESM.pdf]

Additional file 1: Results tables, exploratory analyses and other supporting information

Evaluating the performance of Bayesian and restricted maximum likelihood estimation for stepped wedge cluster randomized trials with a small number of clusters

Kelsey L. Grantham, Jessica Kasza, Stephane Heritier, John B. Carlin, Andrew B. Forbes

A Variance component formulae and implied distributions

Formulae for the cluster and cluster-period variances, σ_C^2 and σ_{CP}^2, respectively, in terms of the primary parameters are given by:

$$\sigma_C^2 = r\sigma_e^2\left(\frac{\rho_1}{1-\rho_1}\right)$$
$$\sigma_{CP}^2 = \sigma_e^2\left(\frac{\rho_1}{1-\rho_1}\right) - \sigma_c^2$$

where $r = \frac{\sigma_C^2}{\sigma_C^2+\sigma_{CP}^2}$ and $\rho_1 = \frac{\sigma_C^2+\sigma_{CP}^2}{\sigma_C^2+\sigma_{CP}^2+\sigma_e^2}$ so that $\frac{\rho_1}{1-\rho_1} = \frac{\sigma_C^2+\sigma_{CP}^2}{\sigma_e^2}$.

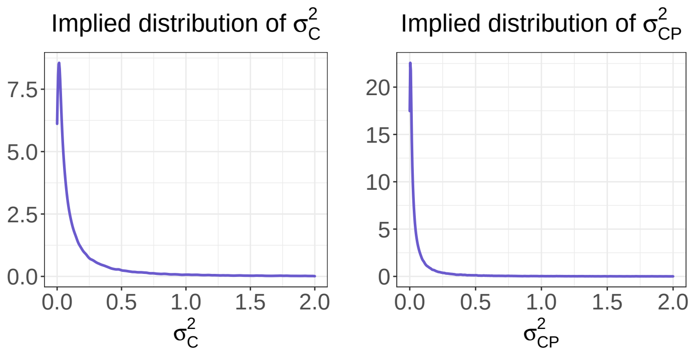

Figure A1: Implied distributions for the cluster and cluster-period variance parameters

B Results tables of performance measures and associated Monte Carlo standard errors

Table B1: Estimated bias ($\times 10,000$) for $\hat{\theta}$ (MCSEs ($\times 10,000$) in parentheses)

				r							
				0.8				1			
ρ_1	T	m	S	Bayesian		REML		Bayesian		REML	
0.05	5	10	1	−37.60	(78)	−43.50	(80)	22.87	(79)	22.03	(79)
			2	33.30	(52)	28.35	(53)	5.40	(54)	− 1.47	(54)
			5	33.14	(35)	29.85	(35)	22.33	(34)	22.51	(34)
		100	1	−32.49	(38)	−37.10	(37)	34.78	(38)	2.20	(25)
			2	3.96	(25)	6.32	(25)	− 8.57	(18)	− 6.81	(18)
			5	16.09	(16)	15.72	(16)	9.08	(12)	9.27	(12)
	9	10	1	−33.75	(40)	−26.28	(40)	−32.90	(38)	−32.15	(38)
			2	24.47	(28)	24.13	(28)	8.05	(26)	9.94	(26)
			5	29.72	(18)	29.13	(18)	13.60	(17)	13.97	(17)
		100	1	21.55	(18)	22.24	(18)	11.52	(13)	14.16	(13)
			2	13.87	(13)	14.14	(13)	10.74	(9)	10.72	(9)
			5	4.96	(8)	4.99	(8)	− 0.44	(6)	− 0.44	(6)
0.1	5	10	1	−39.75	(85)	−42.82	(88)	44.48	(81)	27.37	(82)
			2	38.00	(58)	38.32	(59)	8.82	(56)	6.38	(56)
			5	44.39	(38)	41.38	(38)	23.34	(35)	23.93	(35)
		100	1	−58.17	(46)	−47.96	(46)	− 4.36	(31)	− 0.03	(26)
			2	8.92	(32)	10.63	(32)	− 6.36	(18)	− 7.37	(18)
			5	26.41	(20)	26.38	(20)	9.01	(12)	9.00	(12)
	9	10	1	−37.27	(43)	−32.45	(43)	−22.24	(39)	−24.81	(39)
			2	30.03	(31)	30.12	(31)	5.63	(27)	6.64	(27)
			5	32.10	(20)	31.63	(20)	13.72	(18)	13.63	(18)
		100	1	18.26	(23)	20.09	(23)	12.85	(13)	14.37	(13)
			2	19.95	(16)	20.38	(16)	9.78	(9)	10.26	(9)
			5	7.22	(10)	7.41	(10)	− 0.42	(6)	− 0.53	(6)

Table B2: Estimated MSE ($\times 10,000$) for $\hat{\theta}$ (MCSEs ($\times 10,000$) in parentheses)

				r							
				0.8				1			
ρ_1	T	m	S	Bayesian		REML		Bayesian		REML	
0.05	5	10	1	606	(28.3)	643	(30.9)	597	(27.2)	620	(27.2)
			2	274	(12.3)	282	(12.7)	290	(12.5)	296	(12.8)
			5	122	(5.3)	124	(5.5)	114	(4.7)	115	(4.7)
		100	1	131	(6.6)	136	(6.4)	64	(4.4)	65	(2.9)
			2	63	(2.8)	63	(2.8)	32	(1.6)	32	(1.5)
			5	26	(1.1)	26	(1.1)	13	(0.6)	13	(0.6)
	9	10	1	157	(6.9)	163	(7.3)	140	(6.3)	143	(6.5)
			2	79	(3.6)	80	(3.6)	68	(3.0)	69	(3.0)
			5	33	(1.5)	33	(1.5)	30	(1.3)	30	(1.3)
		100	1	33	(1.5)	33	(1.6)	15	(0.7)	16	(0.7)
			2	17	(0.8)	17	(0.8)	9	(0.4)	9	(0.4)
			5	7	(0.3)	7	(0.3)	3	(0.2)	3	(0.2)
0.1	5	10	1	725	(33.3)	770	(35.7)	637	(29.0)	664	(29.4)
			2	332	(15.0)	343	(15.6)	310	(13.4)	316	(13.8)
			5	145	(6.4)	146	(6.5)	122	(5.0)	122	(5.0)
		100	1	204	(9.9)	214	(10.1)	64	(3.5)	65	(3.0)
			2	100	(4.5)	101	(4.5)	33	(1.6)	33	(1.6)
			5	41	(1.8)	41	(1.8)	14	(0.6)	14	(0.6)
	9	10	1	186	(8.1)	188	(8.3)	149	(6.6)	150	(6.7)
			2	94	(4.2)	95	(4.3)	73	(3.2)	73	(3.2)
			5	39	(1.8)	39	(1.8)	32	(1.4)	32	(1.4)
		100	1	53	(2.4)	53	(2.5)	16	(0.7)	16	(0.7)
			2	27	(1.2)	27	(1.2)	9	(0.4)	9	(0.4)
			5	11	(0.5)	11	(0.5)	3	(0.2)	3	(0.2)

Table B3: Estimated confidence/credible interval coverage for $\hat{\theta}$ (MCSEs in parentheses)

				r					
				0.8			1		
ρ_1	T	m	S	Bayesian	REML	REML (KR)	Bayesian	REML	REML (KR)
0.05	5	10	1	0.967 (0.006)	0.928 (0.009)	0.981 (0.005)	0.951 (0.007)	0.927 (0.008)	0.957 (0.006)
			2	0.967 (0.006)	0.953 (0.007)	0.968 (0.006)	0.945 (0.007)	0.931 (0.008)	0.945 (0.007)
			5	0.954 (0.007)	0.950 (0.007)	0.957 (0.006)	0.962 (0.006)	0.960 (0.006)	0.961 (0.006)
		100	1	0.962 (0.006)	0.919 (0.009)	0.952 (0.007)	0.950 (0.010)	0.943 (0.007)	0.947 (0.007)
			2	0.951 (0.007)	0.938 (0.008)	0.951 (0.007)	0.951 (0.007)	0.949 (0.007)	0.952 (0.007)
			5	0.959 (0.006)	0.955 (0.007)	0.957 (0.006)	0.950 (0.007)	0.948 (0.007)	0.950 (0.007)
	9	10	1	0.959 (0.006)	0.930 (0.008)	0.953 (0.007)	0.958 (0.006)	0.950 (0.007)	0.959 (0.006)
			2	0.948 (0.007)	0.944 (0.007)	0.952 (0.007)	0.963 (0.006)	0.961 (0.006)	0.966 (0.006)
			5	0.948 (0.007)	0.945 (0.007)	0.947 (0.007)	0.949 (0.007)	0.948 (0.007)	0.950 (0.007)
		100	1	0.956 (0.006)	0.949 (0.007)	0.954 (0.007)	0.956 (0.007)	0.952 (0.007)	0.953 (0.007)
			2	0.947 (0.007)	0.944 (0.007)	0.948 (0.007)	0.944 (0.007)	0.948 (0.007)	0.949 (0.007)
			5	0.946 (0.007)	0.947 (0.007)	0.947 (0.007)	0.951 (0.007)	0.952 (0.007)	0.952 (0.007)
0.1	5	10	1	0.955 (0.007)	0.924 (0.009)	0.972 (0.005)	0.943 (0.007)	0.928 (0.008)	0.958 (0.006)
			2	0.961 (0.006)	0.949 (0.007)	0.966 (0.006)	0.943 (0.007)	0.936 (0.008)	0.944 (0.007)
			5	0.954 (0.007)	0.948 (0.007)	0.951 (0.007)	0.958 (0.006)	0.956 (0.006)	0.956 (0.006)
		100	1	0.957 (0.006)	0.918 (0.009)	0.948 (0.007)	0.945 (0.009)	0.946 (0.007)	0.947 (0.007)
			2	0.955 (0.007)	0.940 (0.008)	0.952 (0.007)	0.949 (0.007)	0.950 (0.007)	0.950 (0.007)
			5	0.963 (0.006)	0.957 (0.006)	0.962 (0.006)	0.951 (0.007)	0.951 (0.007)	0.951 (0.007)
	9	10	1	0.952 (0.007)	0.940 (0.008)	0.955 (0.007)	0.957 (0.006)	0.952 (0.007)	0.959 (0.006)
			2	0.949 (0.007)	0.933 (0.008)	0.942 (0.007)	0.962 (0.006)	0.961 (0.006)	0.964 (0.006)
			5	0.947 (0.007)	0.945 (0.007)	0.947 (0.007)	0.951 (0.007)	0.951 (0.007)	0.951 (0.007)
		100	1	0.949 (0.007)	0.941 (0.007)	0.952 (0.007)	0.954 (0.007)	0.957 (0.006)	0.957 (0.006)
			2	0.946 (0.007)	0.940 (0.008)	0.946 (0.007)	0.945 (0.007)	0.944 (0.007)	0.944 (0.007)
			5	0.957 (0.006)	0.956 (0.006)	0.956 (0.006)	0.953 (0.007)	0.954 (0.007)	0.954 (0.007)

Table B4: Estimated relative % error in model-based SE for $\hat{\theta}$ (MCSEs in parentheses)

				r					
				0.8			1		
ρ_1	T	m	S	Bayesian	REML	REML (KR)	Bayesian	REML	REML (KR)
0.05	5	10	1	9.00 (2.5)	−3.19 (2.2)	11.98 (2.6)	0.14 (2.3)	−8.12 (2.1)	1.79 (2.3)
			2	10.42 (2.5)	3.02 (2.3)	9.35 (2.5)	0.05 (2.2)	−4.53 (2.1)	−0.46 (2.2)
			5	1.61 (2.3)	−1.87 (2.2)	0.22 (2.3)	−0.02 (2.2)	−1.75 (2.2)	−0.30 (2.2)
		100	1	11.89 (2.7)	−6.02 (2.2)	0.83 (2.4)	1.47 (3.4)	−1.39 (2.2)	1.44 (2.3)
			2	6.79 (2.4)	−0.32 (2.3)	2.15 (2.3)	1.55 (2.3)	0.07 (2.2)	0.95 (2.3)
			5	1.96 (2.3)	−0.46 (2.2)	0.31 (2.3)	−1.14 (2.2)	−1.41 (2.2)	−1.14 (2.2)
	9	10	1	4.89 (2.4)	−1.85 (2.2)	2.84 (2.3)	4.88 (2.4)	0.50 (2.3)	4.07 (2.3)
			2	2.92 (2.3)	−0.36 (2.2)	1.70 (2.3)	5.62 (2.4)	3.69 (2.3)	5.30 (2.4)
			5	0.21 (2.2)	−1.23 (2.2)	−0.48 (2.2)	0.23 (2.2)	−0.33 (2.2)	0.24 (2.2)
		100	1	3.47 (2.3)	−0.68 (2.2)	0.94 (2.3)	4.79 (2.5)	2.93 (2.3)	3.56 (2.3)
			2	0.42 (2.3)	−1.22 (2.2)	−0.58 (2.2)	−1.37 (2.2)	−1.62 (2.2)	−1.38 (2.2)
			5	−1.68 (2.2)	−2.22 (2.2)	−2.22 (2.2)	1.48 (2.3)	1.39 (2.3)	1.39 (2.3)
0.1	5	10	1	4.45 (2.4)	−4.52 (2.2)	7.42 (2.5)	−1.63 (2.2)	−7.65 (2.1)	−0.44 (2.2)
			2	6.98 (2.4)	1.35 (2.3)	6.15 (2.4)	−0.92 (2.2)	−3.68 (2.2)	−0.87 (2.2)
			5	0.65 (2.3)	−1.88 (2.2)	−0.34 (2.2)	−0.33 (2.2)	−1.07 (2.2)	−0.12 (2.2)
		100	1	7.99 (2.5)	−5.57 (2.2)	0.14 (2.3)	1.39 (2.8)	−0.48 (2.2)	1.11 (2.3)
			2	5.29 (2.4)	−0.08 (2.3)	1.91 (2.3)	0.26 (2.3)	0.19 (2.2)	0.64 (2.3)
			5	2.09 (2.3)	0.10 (2.3)	0.72 (2.3)	−1.27 (2.2)	−1.35 (2.2)	−1.22 (2.2)
	9	10	1	2.87 (2.3)	−0.99 (2.2)	2.36 (2.3)	3.78 (2.3)	1.89 (2.3)	4.18 (2.3)
			2	1.72 (2.3)	−0.45 (2.2)	0.96 (2.3)	4.88 (2.3)	4.13 (2.3)	5.11 (2.4)
			5	−0.73 (2.2)	−1.52 (2.2)	−1.01 (2.2)	0.27 (2.2)	0.04 (2.2)	0.38 (2.2)
		100	1	2.34 (2.3)	−0.63 (2.2)	0.63 (2.3)	3.09 (2.3)	3.36 (2.3)	3.68 (2.3)
			2	1.05 (2.3)	−0.39 (2.2)	0.12 (2.2)	−1.27 (2.2)	−1.38 (2.2)	−1.26 (2.2)
			5	−0.70 (2.2)	−1.13 (2.2)	−1.13 (2.2)	1.34 (2.3)	1.39 (2.3)	1.39 (2.3)

Table B5: Width of estimated confidence/credible intervals for $\hat{\theta}$

				r					
					0.8			1	
ρ_1	T	m	S	Bayesian	REML	REML (KR)	Bayesian	REML	REML (KR)
0.05	5	10	1	1.05	0.95	1.22	0.96	0.89	1.03
			2	0.72	0.68	0.75	0.67	0.64	0.68
			5	0.44	0.43	0.44	0.42	0.41	0.42
		100	1	0.50	0.42	0.50	0.32	0.31	0.32
			2	0.33	0.31	0.33	0.22	0.22	0.22
			5	0.20	0.20	0.20	0.14	0.14	0.14
	9	10	1	0.52	0.49	0.53	0.49	0.47	0.49
			2	0.36	0.35	0.36	0.34	0.34	0.34
			5	0.22	0.22	0.22	0.22	0.21	0.22
		100	1	0.23	0.22	0.23	0.16	0.16	0.16
			2	0.16	0.16	0.16	0.11	0.11	0.11
			5	0.10	0.10	0.10	0.07	0.07	0.07
0.1	5	10	1	1.10	1.03	1.28	0.97	0.93	1.03
			2	0.76	0.73	0.80	0.68	0.67	0.69
			5	0.47	0.46	0.48	0.43	0.43	0.43
		100	1	0.60	0.53	0.62	0.32	0.32	0.32
			2	0.41	0.39	0.41	0.22	0.22	0.23
			5	0.26	0.25	0.26	0.14	0.14	0.14
	9	10	1	0.55	0.53	0.56	0.50	0.49	0.50
			2	0.39	0.38	0.39	0.35	0.35	0.35
			5	0.24	0.24	0.24	0.22	0.22	0.22
		100	1	0.29	0.28	0.29	0.16	0.16	0.16
			2	0.21	0.20	0.21	0.11	0.11	0.11
			5	0.13	0.13	0.13	0.07	0.07	0.07

Table B6: Estimated bias ($\times 10,000$) for $\widehat{\rho_1}$ (MCSEs ($\times 10,000$) in parentheses)

				r							
				0.8				1			
ρ_1	T	m	S	Bayesian		REML		Bayesian		REML	
0.05	5	10	1	280.23	(10)	112.57	(17)	312.05	(10)	28.57	(16)
			2	152.15	(9)	44.10	(11)	165.28	(9)	− 5.24	(12)
			5	60.44	(6)	7.26	(7)	70.81	(6)	− 5.29	(7)
		100	1	122.99	(8)	2.44	(11)	441.43	(12)	4.57	(12)
			2	41.68	(6)	− 7.41	(7)	147.74	(8)	− 5.26	(8)
			5	14.24	(4)	− 6.95	(4)	54.22	(5)	− 6.53	(5)
	9	10	1	111.49	(8)	16.91	(9)	151.58	(9)	− 8.07	(10)
			2	72.40	(6)	16.90	(7)	76.25	(6)	− 8.51	(7)
			5	36.89	(4)	8.51	(4)	36.29	(4)	1.40	(4)
		100	1	26.17	(5)	− 9.42	(7)	163.51	(8)	− 8.28	(8)
			2	17.97	(4)	− 3.45	(5)	67.69	(6)	− 7.28	(5)
			5	10.40	(3)	1.20	(3)	30.45	(3)	0.05	(3)
0.1	5	10	1	23.23	(14)	55.71	(25)	57.17	(13)	− 5.95	(26)
			2	− 0.71	(12)	− 0.38	(16)	27.18	(13)	− 28.00	(18)
			5	− 12.09	(9)	− 21.65	(10)	22.22	(9)	− 15.00	(11)
		100	1	− 28.12	(11)	− 18.39	(19)	231.50	(13)	− 18.95	(22)
			2	− 29.35	(9)	− 23.46	(13)	52.16	(12)	− 22.80	(15)
			5	− 17.58	(7)	− 16.08	(8)	27.16	(8)	− 16.98	(9)
	9	10	1	− 19.71	(11)	− 19.23	(14)	39.16	(12)	− 26.87	(16)
			2	− 0.02	(9)	− 3.26	(10)	27.58	(10)	− 20.25	(11)
			5	4.52	(6)	4.10	(6)	23.82	(7)	0.28	(7)
		100	1	− 41.81	(8)	− 25.16	(12)	63.64	(12)	− 26.65	(15)
			2	− 16.68	(7)	− 10.60	(8)	33.72	(9)	− 18.57	(10)
			5	− 1.31	(5)	0.78	(5)	23.29	(6)	− 1.57	(6)

Table B7: Estimated MSE ($\times 10,000$) for $\widehat{\rho_1}$ (MCSEs ($\times 10,000$) in parentheses)

				r							
				0.8				1			
ρ_1	T	m	S	Bayesian		REML		Bayesian		REML	
0.05	5	10	1	18.0	(0.99)	31.2	(1.90)	18.6	(0.87)	27.2	(1.51)
			2	10.3	(0.56)	12.7	(0.63)	11.5	(0.58)	13.2	(0.66)
			5	4.2	(0.20)	4.5	(0.20)	4.6	(0.24)	4.5	(0.22)
		100	1	6.8	(0.37)	11.6	(0.74)	25.8	(1.29)	14.9	(0.93)
			2	3.7	(0.19)	5.1	(0.24)	8.5	(0.40)	6.8	(0.31)
			5	1.5	(0.07)	1.8	(0.08)	2.9	(0.14)	2.4	(0.11)
	9	10	1	7.4	(0.39)	8.6	(0.47)	9.7	(0.47)	9.2	(0.46)
			2	4.2	(0.20)	4.3	(0.19)	4.8	(0.23)	4.3	(0.19)
			5	1.6	(0.08)	1.7	(0.08)	1.8	(0.09)	1.7	(0.08)
		100	1	3.0	(0.14)	4.5	(0.22)	8.5	(0.43)	6.4	(0.31)
			2	1.7	(0.07)	2.0	(0.09)	3.5	(0.16)	2.9	(0.12)
			5	0.7	(0.03)	0.8	(0.03)	1.2	(0.06)	1.1	(0.05)
0.1	5	10	1	18.3	(0.89)	62.3	(3.18)	17.6	(0.81)	66.9	(3.21)
			2	15.0	(0.63)	27.0	(1.18)	17.1	(0.72)	32.6	(1.44)
			5	7.7	(0.32)	10.0	(0.43)	9.0	(0.42)	11.4	(0.53)
		100	1	11.7	(0.52)	36.0	(2.00)	17.4	(1.08)	46.9	(2.49)
			2	8.3	(0.34)	16.5	(0.72)	14.2	(0.60)	22.4	(0.96)
			5	4.3	(0.18)	5.9	(0.26)	7.1	(0.32)	8.3	(0.37)
	9	10	1	12.0	(0.52)	20.9	(0.99)	15.1	(0.65)	26.1	(1.17)
			2	7.7	(0.32)	10.3	(0.43)	9.7	(0.42)	12.4	(0.54)
			5	3.4	(0.15)	3.9	(0.18)	4.5	(0.20)	4.8	(0.22)
		100	1	7.3	(0.29)	15.2	(0.69)	13.4	(0.61)	21.8	(0.97)
			2	4.7	(0.19)	7.0	(0.29)	8.3	(0.35)	10.1	(0.42)
			5	2.2	(0.09)	2.7	(0.11)	3.6	(0.16)	3.8	(0.16)

Table B8: Estimated bias ($\times 10,000$) for $\hat{r}$ (MCSEs ($\times 10,000$) in parentheses)

ρ_1	T	m	S	Bayesian		REML	
0.05	5	10	1	-581	(12)	-1555	(130)
			2	-573	(18)	-1010	(110)
			5	-493	(21)	-395	(78)
		100	1	-99	(21)	-742	(84)
			2	-135	(24)	-419	(52)
			5	-105	(22)	-176	(29)
	9	10	1	-444	(19)	-597	(89)
			2	-366	(22)	-211	(65)
			5	-186	(23)	57	(44)
		100	1	-84	(24)	-393	(45)
			2	-95	(20)	-211	(27)
			5	-28	(13)	-59	(15)
0.1	5	10	1	-525	(14)	-1018	(110)
			2	-446	(21)	-607	(88)
			5	-274	(23)	-145	(54)
		100	1	-313	(23)	-797	(76)
			2	-300	(24)	-443	(46)
			5	-183	(20)	-176	(25)
	9	10	1	-302	(22)	-375	(68)
			2	-178	(23)	-106	(46)
			5	-22	(21)	24	(29)
		100	1	-269	(24)	-413	(41)
			2	-190	(19)	-207	(25)
			5	-69	(12)	-60	(13)

Table B9: Estimated MSE ($\times 10,000$) for $\hat{r}$ (MCSEs ($\times 10,000$) in parentheses)

ρ_1	T	m	S	Bayesian		REML	
0.05	5	10	1	48	(1.8)	1729	(77.7)
			2	65	(3.5)	1288	(61.8)
			5	69	(3.5)	618	(32.0)
		100	1	41	(1.9)	756	(46.9)
			2	58	(2.8)	285	(20.3)
			5	48	(2.5)	85	(5.1)
	9	10	1	54	(2.6)	822	(41.7)
			2	62	(3.7)	423	(21.5)
			5	55	(3.1)	198	(7.7)
		100	1	59	(2.9)	214	(15.3)
			2	41	(2.7)	76	(6.0)
			5	18	(0.9)	22	(1.1)
0.1	5	10	1	48	(2.1)	1251	(64.0)
			2	65	(3.8)	815	(44.9)
			5	62	(3.4)	297	(16.1)
		100	1	62	(2.7)	634	(42.8)
			2	67	(3.5)	231	(17.6)
			5	42	(2.3)	63	(3.9)
	9	10	1	56	(2.7)	477	(28.0)
			2	57	(3.3)	213	(11.3)
			5	42	(2.1)	81	(3.8)
		100	1	64	(3.4)	186	(13.3)
			2	39	(2.7)	65	(5.1)
			5	15	(0.8)	18	(1.0)

C Session info and package dependencies

```
- Session info ---------------------------------------------------------------
 setting  value
 version  R version 4.0.0 (2020-04-24)
 os       CentOS Linux 7 (Core)
 system   x86_64, linux-gnu
 ui       X11
 language (EN)
 collate  en_AU.UTF-8
 ctype    en_AU.UTF-8
 tz       Australia/Melbourne
 date     2021-05-18

- Packages (rstan) -----------------------------------------------------------
 package         * version    date       lib source
 assertthat        0.2.1      2019-03-21 [1] CRAN (R 4.0.0)
 backports         1.1.9      2020-08-24 [1] CRAN (R 4.0.0)
 BH                1.75.0-0   2021-01-11 [1] CRAN (R 4.0.0)
 callr             3.5.1      2020-10-13 [1] CRAN (R 4.0.0)
 checkmate         2.0.0      2020-02-06 [1] CRAN (R 4.0.0)
 cli               2.3.1      2021-02-23 [1] CRAN (R 4.0.0)
 colorspace        2.0-0      2020-11-11 [1] CRAN (R 4.0.0)
 crayon            1.4.1      2021-02-08 [1] CRAN (R 4.0.0)
 curl              4.3        2019-12-02 [1] CRAN (R 4.0.0)
 desc              1.2.0      2018-05-01 [1] CRAN (R 4.0.0)
 digest            0.6.27     2020-10-24 [1] CRAN (R 4.0.0)
 ellipsis          0.3.1      2020-05-15 [1] CRAN (R 4.0.0)
 fansi             0.4.2      2021-01-15 [1] CRAN (R 4.0.0)
 farver            2.1.0      2021-02-28 [1] CRAN (R 4.0.0)
 ggplot2           3.3.3      2020-12-30 [1] CRAN (R 4.0.0)
 glue              1.4.2      2020-08-27 [1] CRAN (R 4.0.0)
 gridExtra         2.3        2017-09-09 [1] CRAN (R 4.0.0)
 gtable            0.3.0      2019-03-25 [1] CRAN (R 4.0.0)
 inline            0.3.17     2020-12-01 [1] CRAN (R 4.0.0)
 isoband           0.2.4      2021-03-03 [1] CRAN (R 4.0.0)
 jsonlite          1.7.2      2020-12-09 [1] CRAN (R 4.0.0)
 labeling          0.4.2      2020-10-20 [1] CRAN (R 4.0.0)
 lattice           0.20-41    2020-04-02 [1] CRAN (R 4.0.0)
 lifecycle         1.0.0      2021-02-15 [1] CRAN (R 4.0.0)
 loo               2.4.1      2020-12-09 [1] CRAN (R 4.0.0)
 magrittr          2.0.1      2020-11-17 [1] CRAN (R 4.0.0)
 MASS              7.3-53     2020-09-09 [1] CRAN (R 4.0.0)
 Matrix            1.3-2      2021-01-06 [1] CRAN (R 4.0.0)
 matrixStats       0.58.0     2021-01-29 [1] CRAN (R 4.0.0)
 mgcv              1.8-33     2020-08-27 [1] CRAN (R 4.0.0)
```

```
 munsell          0.5.0     2018-06-12 [1] CRAN (R 4.0.0)
 nlme             3.1-152   2021-02-04 [1] CRAN (R 4.0.0)
 pillar           1.5.1     2021-03-05 [1] CRAN (R 4.0.0)
 pkgbuild         1.2.0     2020-12-15 [1] CRAN (R 4.0.0)
 pkgconfig        2.0.3     2019-09-22 [1] CRAN (R 4.0.0)
 prettyunits      1.1.1     2020-01-24 [1] CRAN (R 4.0.0)
 processx         3.4.4     2020-09-03 [1] CRAN (R 4.0.0)
 ps               1.3.4     2020-08-11 [1] CRAN (R 4.0.0)
 R6               2.5.0     2020-10-28 [1] CRAN (R 4.0.0)
 RColorBrewer     1.1-2     2014-12-07 [1] CRAN (R 4.0.0)
 Rcpp             1.0.6     2021-01-15 [1] CRAN (R 4.0.0)
 RcppEigen        0.3.3.9.1 2020-12-17 [1] CRAN (R 4.0.0)
 RcppParallel     5.0.2     2020-06-24 [1] CRAN (R 4.0.0)
 rlang            0.4.10    2020-12-30 [1] CRAN (R 4.0.0)
 rprojroot        2.0.2     2020-11-15 [1] CRAN (R 4.0.0)
 rstan            2.21.2    2020-07-27 [1] CRAN (R 4.0.0)
 scales           1.1.1     2020-05-11 [1] CRAN (R 4.0.0)
 StanHeaders      2.21.0-7  2020-12-17 [1] CRAN (R 4.0.0)
 tibble           3.1.0     2021-02-25 [1] CRAN (R 4.0.0)
 utf8             1.2.1     2021-03-12 [1] CRAN (R 4.0.0)
 V8               3.4.0     2020-11-04 [1] CRAN (R 4.0.0)
 vctrs            0.3.6     2020-12-17 [1] CRAN (R 4.0.0)
 viridisLite      0.3.0     2018-02-01 [1] CRAN (R 4.0.0)
 withr            2.4.1     2021-01-26 [1] CRAN (R 4.0.0)

- Packages (lme4) ---------------------------------------------------------------
 package      * version   date       lib source
 boot           1.3-26    2021-01-25 [1] CRAN (R 4.0.0)
 lattice        0.20-41   2020-04-02 [1] CRAN (R 4.0.0)
 lme4           1.1-26    2020-12-01 [1] CRAN (R 4.0.0)
 MASS           7.3-53    2020-09-09 [1] CRAN (R 4.0.0)
 Matrix         1.3-2     2021-01-06 [1] CRAN (R 4.0.0)
 minqa          1.2.4     2014-10-09 [1] CRAN (R 4.0.0)
 nlme           3.1-152   2021-02-04 [1] CRAN (R 4.0.0)
 nloptr         1.2.2.2   2020-07-02 [1] CRAN (R 4.0.0)
 Rcpp           1.0.6     2021-01-15 [1] CRAN (R 4.0.0)
 RcppEigen      0.3.3.9.1 2020-12-17 [1] CRAN (R 4.0.0)
 statmod        1.4.35    2020-10-19 [1] CRAN (R 4.0.0)
```

D Exploratory analysis comparing valid with invalid Bayesian replicates

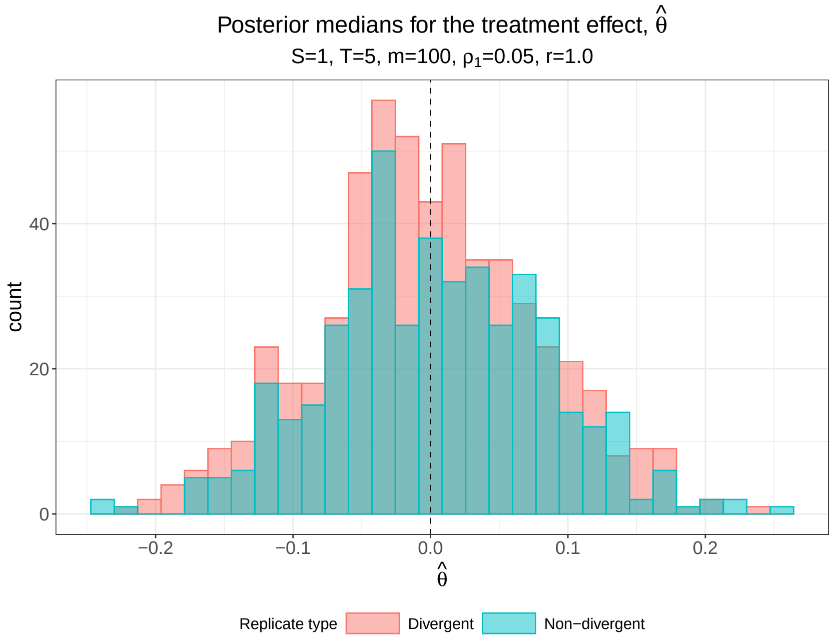

Figure D1: Histograms of marginal posterior medians for $\hat{\theta}$ for the 558 invalid replicates yielding divergent transitions after warmup (red) and 442 valid replicates without any divergent transitions (blue), for the trial configuration with one cluster per treatment sequence, 5 periods, 100 subjects per cluster-period and with a within-period intracluster correlation of 0.05 and a cluster autocorrelation of 1.0. Note that the shading of the bars is semi-transparent to show overlap between the histograms

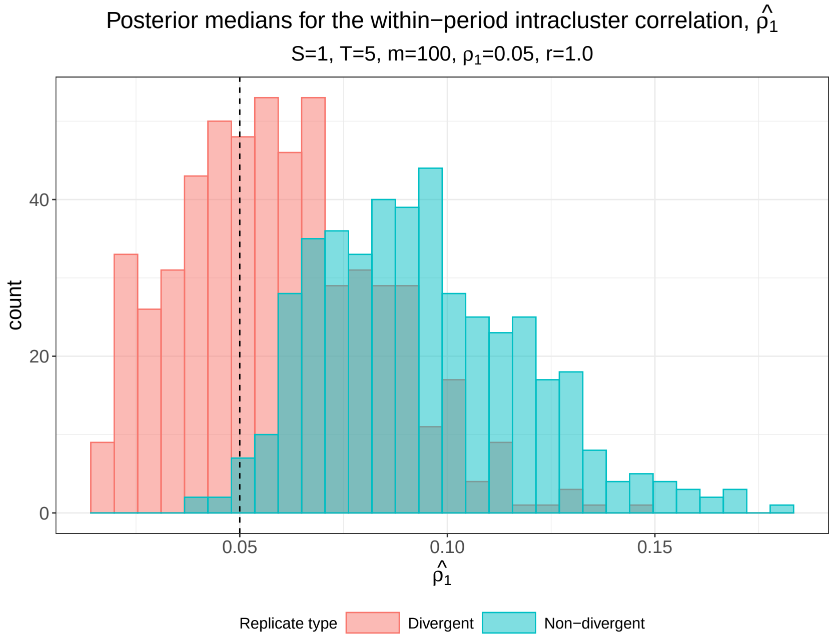

Figure D2: Histograms of marginal posterior medians for $\widehat{\rho_1}$ for the 558 invalid replicates yielding divergent transitions after warmup (red) and 442 valid replicates without any divergent transitions (blue), for the trial configuration with one cluster per treatment sequence, 5 periods, 100 subjects per cluster-period and with a within-period intracluster correlation of 0.05 and a cluster autocorrelation of 1.0. Note that the shading of the bars is semi-transparent to show overlap between the histograms

E Exploratory analysis comparing results with different treatment effect values

Table E1: Estimated bias ($\times 10,000$) for $\hat{\theta}$ (MCSEs ($\times 10,000$) in parentheses), for different treatment effect values, for trial configurations with one cluster per treatment sequence, 5 periods and 10 subjects per cluster-period

		r							
		0.8				1			
ρ_1	θ	Bayesian		REML		Bayesian		REML	
0.05	0	-37.6	(78)	-43.5	(80)	22.9	(79)	22.0	(79)
	10	-36.3	(78)	-43.5	(80)	10.5	(79)	22.0	(79)
	100	-42.3	(78)	-43.5	(80)	-6.6	(78)	22.0	(79)
0.1	0	-39.7	(85)	-42.8	(88)	44.5	(81)	27.4	(82)
	10	-38.8	(85)	-42.8	(88)	27.8	(81)	27.4	(82)
	100	-50.3	(85)	-42.8	(88)	42.1	(80)	27.4	(82)

Table E2: Estimated MSE ($\times 10,000$) for $\hat{\theta}$ (MCSEs ($\times 10,000$) in parentheses), for different treatment effect values, for trial configurations with one cluster per treatment sequence, 5 periods and 10 subjects per cluster-period

		r							
		0.8				1			
ρ_1	θ	Bayesian		REML		Bayesian		REML	
0.05	0	606	(28)	643	(31)	597	(27)	620	(27)
	10	606	(28)	643	(31)	590	(26)	620	(27)
	100	607	(28)	643	(31)	586	(27)	620	(27)
0.1	0	725	(33)	770	(36)	637	(29)	664	(29)
	10	722	(33)	770	(36)	638	(29)	664	(29)
	100	722	(33)	770	(36)	627	(29)	664	(29)
